# Medicines Received

**Published:** 1868-10

**Authors:** 


MEDICINES RECEIVED.—We have received from Hegeman & Co., of New York, some specimens of cod liver oil, which seem to us of unsurpassed quality. This oil has been in our market for some years and has been generally regarded as the best the market affords. Hegeman & Co. also send Elixir of Bark and Iron, which for elegance of preparation and value of composition are unsurpassed. We have also to acknowledge receipt of various other similar preparations, all of which are elegantly compounded.

